# Survey for Adventive Populations of the Samurai Wasp, *Trissolcus japonicus* (Hymenoptera: Scelionidae) in Pennsylvania at Commercial Fruit Orchards and the Surrounding Forest

**DOI:** 10.3390/insects12030258

**Published:** 2021-03-19

**Authors:** Hillary M. Peterson, Elijah Talamas, Grzegorz Krawczyk

**Affiliations:** 1 Fruit Research and Extension Center, Department of Entomology, Pennsylvania State University, Biglerville, PA 17307-0330, USA; gxk13@psu.edu; 2 Florida Department of Agriculture and Consumer Services, Division of Plant Industry, Gainesville, FL 32608, USA; Elijah.Talamas@fdacs.gov

**Keywords:** Hymenoptera, Scelionidae, Hemiptera, Pentatomidae, yellow sticky card, invasive species

## Abstract

**Simple Summary:**

Invasive species that are freed of associated natural predators increase in population and disrupt the management plans of growers. The brown marmorated stink bug, *Halyomorpha halys,* is an invasive species that originated in Asia. A promising management tactic for the species is to release an associated parasitoid, the samurai wasp, *Trissolcus japonicus*. Populations of the samurai wasp have begun to adventively establish in several regions, including Pennsylvania. In order to monitor and use the species as an alternative management strategy to insecticides, it is imperative to understand the baseline populations during the early establishment phase. The aims of this study were to determine if the samurai wasp is already present in commercial orchards in Pennsylvania, where the brown marmorated stink bug has been present in high numbers since approximately 2010. Native *Trissolcus* wasps were also identified. This study found the samurai wasp in eight counties in Pennsylvania with several orchards containing populations of the species within orchard blocks. These baseline data provide a starting point for controlling *H. halys* naturally, which was previously controlled only with broad-spectrum insecticides.

**Abstract:**

The samurai wasp, *Trissolcus japonicus* (Ashmead) (Hymenoptera: Scelionidae), is an egg parasitoid associated with the brown marmorated stink bug, *Halyomorpha halys* (Stål) (Hemiptera: Pentatomidae). *Trissolcus japonicus* is a candidate for classical biological control of *H. halys* populations. Since 2014, adventive populations of *T. japonicus* have been detected in 14 US states, in the Canadian provinces of British Columbia and Ontario, and in two European countries, Switzerland and Italy. Establishing baseline information about populations of *T. japonicus* is important, as this species is not host specific to *H. halys* and the potential ecological effects of the accidental introductions are not fully known. In this study, yellow sticky cards were deployed at commercial fruit orchards in nine counties in Pennsylvania separated by more than 400 km. *Trissolcus japonicus* was detected on cards in eight counties, and in two habitats, in the orchard and at the forest border. Other native species of Scelionidae known to attack the eggs of *H. halys* were also identified, including *Trissolcus euschisti* (Ashmead), *Trissolcus brochymenae* (Ashmead), and *Telenomus podisi* Ashmead (Hymenoptera: Scelionidae). These results are important baseline ecological knowledge for both *T. japonicus*, which appears to be established in orchards throughout Pennsylvania, and other native Scelionidae.

## 1. Introduction

The brown marmorated stink bug, *Halyomorpha halys* (Stål) (Hemiptera: Pentatomidae), is an invasive species in many regions worldwide, and has become a fascinating case study for potential classical biological control. *Halyomorpha halys* is estimated to have first arrived in North America during the mid-1990s [1]. However, populations did not increase to a severe economic outbreak status before 2008–2010, when, in the Mid-Atlantic region, some stone fruit growers lost over 90% of their crop in a single year [2]. *Halyomorpha halys* is native to China, South Korea, Taiwan and Japan, where it is an occasional pest of many crops [3]. Crops damaged by adults and nymphs include fruits such as apple, peach, and pear, and vegetables such as cucumber, eggplant, and sweet corn [2].

In Asia, *H. halys* is partially controlled through applications of pyrethroid and neonicotinoid insecticides [3], and by natural enemies. A variety of natural enemies, including predators, pathogens and parasitoids, provide biological control for all life stages of *H. halys* [3,4]. Studies were conducted to determine if classical biological control (importation of associated natural enemies) might be an option for regions where *H. halys* had invaded. Collections of wild *H. halys* egg masses in Asia identified two promising potential classical biological control agents: *Trissolcus japonicus* (Ashmead) and *Trissolcus cultratus* (Mayr) [5]. *Trissolcus japonicus* is particularly effective in Asia, with an average annual parasitism rate of 50% [6]. In addition to *T. japonicus,* a recent study has identified another adventive population of a species of from Asia in Italy, *Trissolcus mitsukurii* (Ashmead) which also has a higher parasitism rate than the parasitoids native to the region [7]. To understand the context in which a classical biological control agent would potentially be released, studies were conducted to understand the biodiversity of native pentatomid egg parasitoids in invaded regions. These surveys were necessary, as native species may have the capacity to switch to *H. halys* as a host, and *T. japonicus* releases represent potential risk because it is not host-specific to *H. halys* [8,9,10]. A review of 98 *H. halys* egg mass studies conducted in North America and Europe identified hymenopterans in the families Scelionidae, Eupelmidae, and Encyrtidae as the most commonly detected egg parasitoids [11]. Unfortunately, most of these studies indicated low rates of parasitism (<5%), emphasizing the importance of potential classical biological control releases of more effective natural enemies.

While quarantine host-range and behavioral studies were being conducted on *T. japonicus* and *T. cultratus* [5,8,12], adventive populations of *T. japonicus* were discovered in North America in 2014 from fresh and frozen *H. halys* egg masses deployed in a wooded plot in Maryland [13]. This discovery led to further surveys for *T. japonicus* in varying habitats of *H. halys* invaded regions, predominantly using sentinel or naturally occurring *H. halys* eggs. Sentinel *H. halys* egg mass studies detected *T. japonicus* in wooded habitats in Beltsville, MD (USA) [14], a wooded suburban area in southcentral Vancouver, WA (USA) [15], ornamental trees in Portland, OR (USA) [12], apple orchards in the municipalities of Bellinzona and Manno, Canton of Ticino, Switzerland [16], a suburban setting in Chilliwack, BC, Canada [11], an organic farm with mixed crops including peaches, raspberries and grapes, ornamental trees in Holt MI (USA) [17], and in a peach orchard in Richwood, NJ (USA) [18]. Collections and rearing of wild *H. halys* egg masses detected *T. japonicus* in wooded suburban Vancouver, WA (USA) [15], from the mid-canopy of *Ailanthus altissima* growing on the edge of the woods of three commercial orchards near Winchester, VA (USA) [19], from *Catalpa speciosa* in two suburban locations in Salt Lake City, UT, USA [20], and in a parking lot on *Acer campestris* trees in northern Italy [21]. Finally, yellow sticky cards detected *T. japonicus* while deployed in the mid and upper canopies of *A. altissima* on the edge of three commercial orchards near Winchester, VA (USA) [22], and yellow sticky cards were also successfully used to detect *T. japonicus* in redistribution trials in 22 sites within the Columbia Plateau, Willamette Valley, Eastern Cascades Slopes, and Rogue Valley, OR, USA [23]. A recent *H. halys* sentinel egg mass survey of vegetable plots, fruit orchards, and wooded habitats in Byron, Watkinsville, Union Point, Ellijay, Blue Ridge, Cleveland, Athens, Monroe, and Williamson, GA (USA) and in Auburn, Clanton, Tuscaloosa, Prattville and Shorter, AL (USA) did not find *T. japonicus* [24], indicating that the species may not yet be established in much of the southern United States.

In this project, we used yellow sticky cards to characterize populations of *T. japonicus* and native scelionid parasitoids known to attack *H. halys* in commercial and research orchards in Pennsylvania with historical presence of *H. halys* populations. This study was conducted after a preliminary study using yellow sticky cards and sentinel *H. haly*s egg masses in 2017 detected the first records of *T. japonicus* for Pennsylvania in Lancaster and Adams counties. Our objectives were the following: (1) determine the distribution of *T. japonicus* in Pennsylvania; (2) compare populations of *T. japonicus* with native egg parasitoids known to attack *H. halys*; (3) compare species compositions of parasitoids across three habitats (orchard, forest, and forest border) and throughout the entire season when *H. halys* adults are present; (4) further develop effective sampling protocols for Scelionidae using yellow sticky cards.

## 2. Materials and Methods

### 2.1. Survey Locations

We surveyed at fruit orchards in Pennsylvania with historically high populations of *H. halys*. In 2018, we sampled four orchards in four counties, and in 2019, we sampled nine orchards in eight counties. Three of the four locations from 2018 (Berks location 1, Allegheny, and Centre counties) were sampled again in 2019, and an additional location within Berks county (Berks location 2) was sampled in 2019 due to high prevalence of *H. halys* in the previous year. In total, ten orchards were sampled in nine counties across both years (Table 1). All orchards were conventionally managed, with several employing intensive integrated pest management practices including pest monitoring and mating disruption, except for one that was classified as an experimental orchard (Centre County). Orchards ranged in size from 16–202 ha and, generally, the area sampled was smaller than the fruit-growing portion of the orchard, such as sampling taking place in and surrounding only 1 to 4 blocks of fruit. Sampling areas in each orchard ranged in size from 5–58 ha. All surveyed orchards predominantly grew apples, and also peaches, plums, nectarines, and pears.

### 2.2. Deployment of Yellow Sticky Cards (YSC)

We deployed yellow sticky cards (YSC) (double-sided, 20 × 14 cm, Alpha Scents, Inc., West Linn, OR, USA) baited with 1 package of *H. halys* pheromone dual lures (Trécé, Inc., Adair, OK, USA; affixed to the top of the card; replaced at 12-week intervals). YSC were deployed during the ovipositional period of *H. halys* typically observed in Pennsylvania, generally from mid-June through the end of August (Krawczyk, unpublished data). Due to constraints on time and card availability, intervals and sampling methods differed between locations and years. Of the four orchards we sampled in 2018, one orchard (Berks County 1) was sampled from 5 June to 15 August at an average of 10-day intervals. All other orchards sampled in 2018 were surveyed from 25 July to 20 October using YSC deployed for an average of 43-day intervals. Of the nine orchards sampled in 2019, one orchard (Berks County 1) was sampled from the weeks of 23 May to 10 October in two sampling intervals (50 and 90 days). The other orchards sampled in 2019 were surveyed from the weeks of 15 May to 1 September for three intervals averaging 36 days. Sampling location and habitat details are provided in Table 1. All sampling dates and intervals are provided in Supplementary Material 1.

The YSC were deployed at approximately 1.8 m height in vegetation or occasionally onto deer fencing when vegetation was not available. YSC were deployed using two twist ties (Trécé Inc., Adair, OK, USA) affixed to substrate to prevent the loss of cards due to wind. The majority of YSC were deployed with both sticky sides exposed; however, only one-sided YSC were deployed for 9 of the 10 deployment intervals at the Berks County 1 location in 2018. Depending on availability of habitats, we surveyed one to three habitats in each orchard: “orchard”, “forest border”, and “forest” (Table 1). In the orchard habitat, cards were placed in the middle of a block of tree fruit, with the majority of deployments in apples, and the rest in peaches and pears. The forest habitat had vegetation in the woods surrounding orchard blocks, 20–100 m depth from the border of the orchard with the majority of deployment locations in sassafras (*Sassafras albidum*), tree of heaven (*Ailanthus altissima*), and deer fencing present in the woods. The forest border habitat was the outer edge of the forested sites facing the orchard blocks, with the majority of deployment locations on maple (*Acer* sp.), oak (*Quercus* sp.), sumac (*Rhus* sp.), tree of heaven (*A. altissima*), or deer fencing.

### 2.3. Collection and Identification of Parasitoids

Upon completion of the sampling interval, YSC were collected into receptacles with space between each card to prevent self-adhesion. To identify *T. japonicus* and other Scelionidae, cards were scanned under a dissection microscope (SMZ 745T, Nikon, Tokyo, Japan). Parasitoids were removed from cards with a hole punch (McGill 2” Reach Punchline Hole Punch, ¼ inch round, Advantus Corp., Jacksonville, FL, UAS), and these sections were either affixed inside of Petri dishes using double-sided tape (Scotch, 3M**^®^**, St. Paul, MN, USA) or placed in a dish of histological clearing agent (Histo-Clear II, National Diagnostics, Atlanta, GA, USA) to dissolve the glue, stored in 95% ethanol, and point mounted for identification. Placement of sections into Petri dishes allowed for identifications of several specimens at one time and easy mailing of specimens. The use of Histo-Clear allowed for immediate identification of specimens whose diagnostic characters were obscured by glue. Identifications for *Trissolcus* spp. followed Talamas et al. [5], and *Telenomus* spp. followed Johnson [25]. Parasitoids that were not identifiable to species or are not confirmed to attack *H. halys* eggs in Pennsylvania were classified as “other Scelionidae”. These included *Trissolcus edessae* Fouts, *Trissolcus thyantae* Ashmead, *Telenomus cristatus* Johnson, *Telenomus goliathus* Johnson, and *Telenomus persimilis* Ashmead. Full count data is provided in Supplementary Material 1.

### 2.4. Statistical Analysis

The combined total of all Scelionidae captured at each habitat (orchard, forest border, and forest) and within each part of the season (early season = May and June; middle season = July and August; late season = September and October) in 2018 and 2019 were analyzed using a generalized linear mixed model with a negative binomial distribution. We included orchard (county) as the random factor, and number of days the YSC was exposed as an offset value. The Tukey’s method for comparing estimated marginal means was used to compare values with significant differences across the treatments. Statistical analyses did not include the 2018 data for Berks (1), as these YSC were deployed at drastically different sampling intervals with only one side of the cards exposed. The model did not have enough power to statistically compare individual species of parasitoids. All statistical analyses were conducted in R version 1.1.463 [26]. Generalized linear mixed models were fit using *lme4* package (*glmer.nb*) [27], and post-hoc analyses were conducted using the Tukey method for comparing estimated marginal means from package *emmeans* (*emmeans*) [28]. Exemplary data code may be obtained online (Supplementary Material 2).

## 3. Results

**Figure 1:**
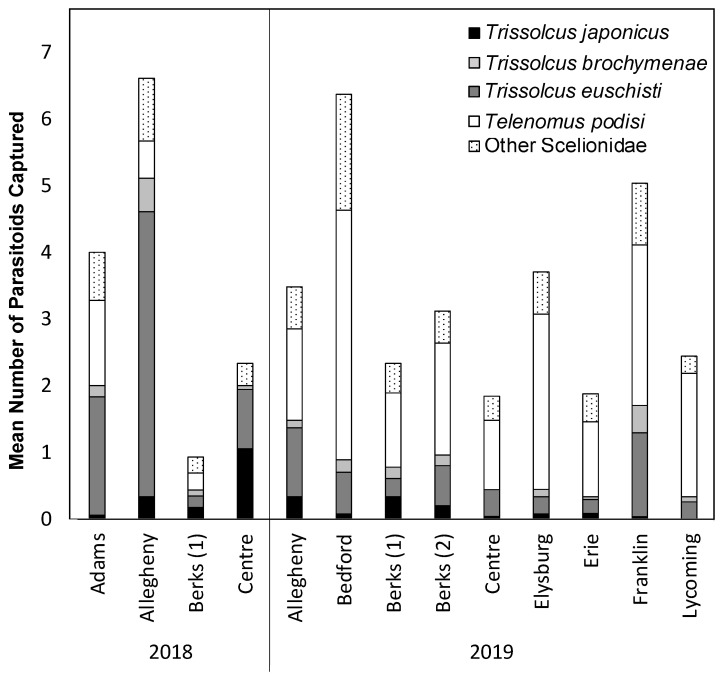
Mean number of *Trissolcus japonicus* and other known scelionid egg parasitoids captured in across the entire season and all orchards sampled during the 2018 and 2019 seasons.

We report the first detections of *T. japonicus* in three counties in Pennsylvania in 2018, and in five counties in Pennsylvania in 2019 (Table 1), with a total of ten counties with at least one detection as of 2019. The initial detection of *T. japonicus* in Pennsylvania occurred in 2017 as a part of a study that used both *H. halys* sentinel egg masses and yellow sticky cards in Adams and Lancaster counties (Peterson et al, in prep). Across both years of this study (2018–2019), *T. japonicus* was found on YSC deployed in commercially managed apple, peach, and pear orchards (Table 1). These detections were both in orchard habitats and at the forest border between the orchard and forest habitat on vegetation, but never in the forest (Table 1). In addition to *T. japonicus,* we also collected Scelionidae native egg parasitoids known to attack *H. halys*, including *Trissolcus euschisti*, *Trissolcus brochymenae*, *Telenomus podisi*, as well as small numbers of other parasitoids known to attack native Pentatomidae at all locations sampled (Figure 1, Supplementary Material 1).

**Figure 2:**
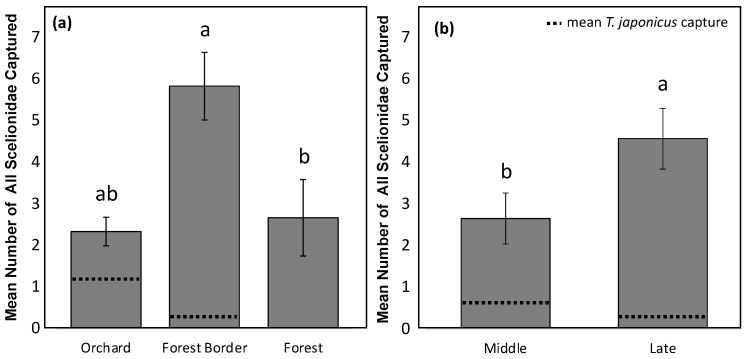
Mean number of all Scelionidae parasitoids captured in 2018 on YSC in (**a**) three habitats (orchard, forest border, and forest) across the entire season at all orchards sampled (except for Berks (1)), and in (**b**) in the middle season (July and August) and late season (September and October) across three entire habitats at all orchards sampled (except for Berks (1)). Letters compare means for habitats (*X*^2^ = 12.47, df = 2, *p* = 0.002) and season (*X*^2^ = 4.37, df = 1, *p* = 0.037), with dotted lines representing *T. japonicus* capture. Berks (1) was not included in the analysis due to a differing sample method.

In 2018, total Scelionidae capture on YSC differed by both habitat (*X*^2^ = 12.47, df = 2, *p* = 0.002), with the highest capture at the forest border (Figure 2a) and season (*X*^2^ = 4.37, df = 1, *p* = 0.037), with the highest captures in the late season (September and October) (Figure 2b). The trend for *T. japonicus* did not always follow the overall trend for native Scelionidae, however, with numerically more captures in the orchard habitat and in the middle of the season (July and August) (Figure 2).

**Figure 3:**
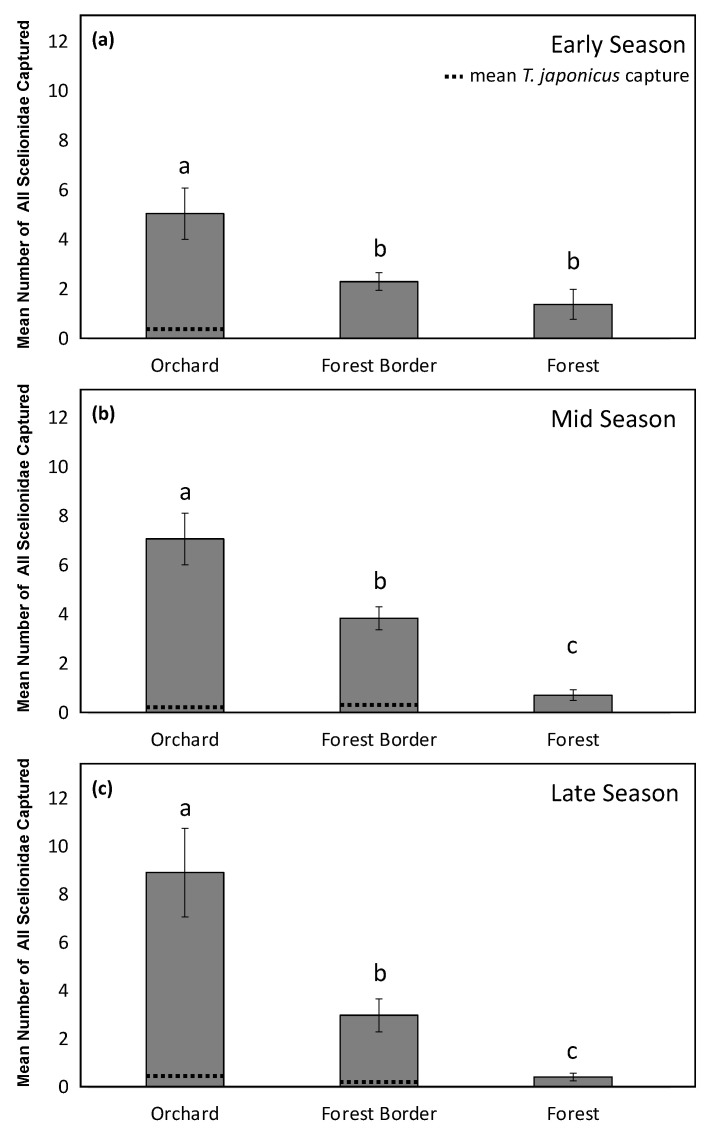
Mean number of all Scelionidae parasitoids captured in 2019 on YSC in the (**a**) early season (May and June) (**b**) middle season (July and August) and (**c**) late season (September and October) at three habitats (orchard, forest border, and forest) at all orchards sampled. Letters compare means for the early (*X*^2^ = 16.81, df = 2, *p* < 0.001), middle (*X*^2^ = 53.42, df = 2, *p* < 0.001), and late season (*X*^2^ = 41.15, df = 2, *p* < 0.001), with dotted lines representing *T. japonicus* capture.

In 2019, there was an interaction between the habitat and the season for the analyses of total Scelionidae captured on YSC (*X*^2^ = 9.75, df = 4, *p* = 0.045). Analyzing the differences between Scelionidae capture at each habitat within each season, we found more Scelionidae at the orchard habitat than at both the forest border and the forest in the early season (*X*^2^ = 16.81, df = 2, *p* < 0.001) (Figure 3a), and the highest capture of all Scelionidae in the orchard habitat and lowest at the forest habitat in both the middle (*X*^2^ = 53.42, df = 2, *p* < 0.001), and late (*X*^2^ = 41.15, df = 2, *p* < 0.001) season (Figure 3b,c). In contrast with 2018, *T. japonicus* captures generally followed the same habitat trends, and were found throughout the entire season (Figure 3).

## 4. Discussion

In this study, we report the presence of a total of 69 individual *T. japonicus* detected across two years and nine orchards located in Pennsylvania. In addition to describing populations of *T. japonicus,* we also determined that YSC could also be used to identify populations of other scelionid species present at the same locations. Yellow sticky cards have recently been demonstrated to be an effective and less time-consuming tool for the detection of *T. japonicus* when compared with the deployment and rearing time needed for sentinel egg masses [22]. However, all discoveries of adventive *T. japonicus* thus far have utilized only sentinel or wild collected *H. halys* eggs (e.g., [14,15,16,17]). Identifying other known parasitoids at the same locations as adventive populations, during a year likely close to the initial introductions of the species, provides context which can be compared in later years as *T. japonicus* populations possibly establish further. These data may be used to construct qualitative food webs, which are important for comparing and predicting further ecological relationships [28].

The trends in detections of *T. japonicus* did not consistently follow the detection trends of other native species of Scelionidae. Generally, average *T. japonicus* detections at each location accounted for <20% of the species of Scelionidae captured on cards. There was one exception to this trend, however, where *T. japonicus* accounted for 45% of Scelionidae captured at the Centre county location in 2018, where only the orchard habitat was surveyed. This percentage drastically decreased to 2% at the same location in 2019, when YSC were split amongst the three habitats instead of only the orchard habitat. Generally, across all locations surveyed, *T. japonicus* detections were higher in the orchard habitat when surveyed in both 2018 and 2019. Other studies have detected *T. japonicus* in similar habitats using sentinel *H. halys* egg masses, including the border of peaches, grapes, and raspberries at an experimental farm in Michigan [17], in peaches, but not apples, in New Jersey [18], and in commercial apples in Switzerland [16]. The detection of *T. japonicus* in apple, peach, and pear blocks in the current study is important information for growers when implementing integrated pest management that protects *T. japonicus* as a biocontrol agent. A recent study demonstrated high sensitivity of *T. japonicus* to pyrethroids and neonicotinoids and the use of these chemicals may ultimately be detrimental to controlling *H. halys* [29].

In contrast, during both years of the study, *T. japonicus* was never detected on YSC deployed in the forest habitat, while populations of native Scelionidae were discovered at comparable rates in the orchard in 2018, and at comparable rates at the forest border in the early season of 2019. It is possible the YSC deployed in this study, at an approximate height of 1.5 m, were not elevated high enough in the forest canopy to intercept *T. japonicus.* It has been demonstrated that *T. japonicus* are more likely to be detected from wild-laid *H. halys* egg masses in the upper two-thirds of the canopy of female tree of heaven (*A. altissima*) bordering orchards [19], and on YSC in canopies deployed in the middle canopy of female tree of heaven at 4.8 m. While it would potentially have been advantageous for this study to have surveyed at higher altitudes in the forest, preliminary work by our group in smaller trials demonstrated that it would be very time consuming to deploy cards in this manner to monitor the number of sites used in this study (Peterson et al *in prep*). Two studies using sentinel *H. halys* egg masses have detected the species in forested areas in Maryland [14] and in a lightly wooded public recreation area in Washington [15], demonstrating potential differences in the ability to detect the *T. japonicus* employing methods with differing visual and olfactory stimuli. It would be beneficial for populations of *T. japonicus* to become established in forested habitats, as *H. halys* often reside in these habitats surrounding orchards. This habitat thus serves as a refuge where *H. halys and T. japonicus* can be protected from insecticidal treatments in the orchard.

Differences in detection trends across the seasons were also present when comparing *T. japonicus* with native Scelionidae. In 2018, all Scelionidae were found at a higher rate during the late season; however, *T. japonicus* captures were the highest in the middle of the season. In 2019, there was an interaction between habitat and seasonality for all Scelionidae, demonstrating higher captures in the later seasons with a more divided capture rate amongst habitats in the middle and late seasons than the early season. These trends were in contrast with *T. japonicus*, which was detected with a more drastic habitat “split” in the early season, found only in the orchard habitat, than the middle and late season, where the species was detected somewhat evenly in both the orchard and forest border habitats. While these contrasts point to potential differences in habitat and seasonal preference between *T. japonicus* and native Scelionidae, which may have to do with preference to *H. halys* and other native Pentatomidae habitat preferences, further studies would be needed for clarification.

As was described in Quinn et al. [22], YSC are a useful addition to the “tool box” for describing populations of egg parasitoids of *H. halys*, and possibly other species. This study demonstrates that YSC allow for a wide survey for *T. japonicus* in space, with approximately 400 km between the furthest sampling locations in Pennsylvania, and time, with successful detections on cards passively sampling for as long as 90 days. Unfortunately, drawbacks to using YSC also exist. Many replicates of cards in this study were lost either to strong winds or rain or were made unusable by debris blowing onto the card. YSC also produce considerable insect bycatch, and occasionally large clusters of bird feathers were found affixed to cards. In addition, identification of some organisms to species level requires removal of the glue with solvent. Finally, not all groups of insects can be identified to species on YSC. Soft bodied parasitoids in the superfamily Chalcidoidea, such as *Ooencyrtus* spp. and *Anastatus* spp. are known parasitoids of *H. halys* but were generally too damaged to identify on the cards in this study due to long sampling windows and exposure to the environmental factors. These drawbacks highlight a benefit to using sentinel, or wild-collected egg masses, which result in live specimens that can be used for creating parasitoid colonies or placed directly into ethanol for preservation and processing for identification. When possible, utilizing both methods in a sampling area would provide the most useful information.

In this study, long intervals were necessary to facilitate deploying and retrieving YSC placed in orchards with large distances between them. By “trading” cards which had been deployed for long intervals, rather than sampling over shorter intervals spread throughout the season, we were able to reduce the travel for the three sets by two trips per location. Long sampling intervals do have drawbacks as well. For example, the precision in sampling phenology is lost, and increased sampling intervals also contribute to degradation of cards and specimens. A major benefit of conducting long sampling intervals in this study is the demonstration that the specimens were not too degraded to allow the identification. This may be important for future studies which are constrained financially or by time. It may be possible to use other means to reduce travel across large areas, however, such as collaborations with growers through, perhaps, mailing YSC to many locations.

It remains unknown how *T. japonicus* populations have established across such a wide variety of regions and habitats in North America, Canada, and Europe. Explorations of the dispersal and overwintering behaviors of the species may be possible by implementing a variety of sampling methods, including YSC. Future studies should investigate the optimal sampling window for YSC deployed under different temperature conditions, and the feasibility of utilizing additional insect trapping methods, such as yellow pan traps or Lindgren funnel traps for *T. japonicus*, which are less destructive to specimens but require more labor. The presence of *T. japonicus* in regions where *H. halys* has been established is promising in light of *H. halys* being an economical pest, but also must be understood as a potential risk to be monitored and assessed in the future. *Trissolcus japonicus* is not host-specific. Host-range studies have demonstrated the ability of *T. japonicus* to develop in six Pentatomidae native to California in no-choice, and sometimes binary-choice laboratory experiments [10], and eleven Pentatomidae native to Europe in no-choice laboratory experiments [30]. This is not unexpected, as in northern China, a recent study demonstrated the ability of *T. japonicus* to develop in the eggs of seven species of Pentatomidae in no-choice laboratory experiments, and was reared from field-collected eggs of two non-target Pentatomidae [31]. Due to the ability of *T. japonicus* to develop on non-target hosts, it has the potential to disrupt relationships between native parasitoids and stink bugs in unpredicted ways.

In summary, this study confirms the presence of *T. japonicus*, along with population data for native Scelionidae using YSC in commercial orchards across the state of Pennsylvania, providing baseline data for a species that has future potential to control a damaging invasive pest species through natural and sustainable biological control.

## Figures and Tables

**Table 1:** *Trissolcus japonicus* (N = total number) detections on yellow sticky cards (n = number of cards deployed and retrieved) at three habitats sampled in four (2018), and at eight (2019) Pennsylvania counties at and around fruit orchards.

Year	County (Location)	N (n) per Habitat			Tree Fruit with *T. japonicus*
		Orchard	Forest Border	Forest	
2018	Berks (1)	13 (28)	2 (29)	0 (30)	Apple, Peach
	Adams	—	1 (18)	—	—
	Allegheny	—	6 (12)	0 (6)	—
	Centre	19 (18)	—	—	Apple
2019	Bedford	0 (9)	2 (12)	0 (6)	—
	Berks (1)	5 (6)	1 (4)	0 (8)	Apple, Peach
	Northumberland	2 (9)	0 (9)	0 (9)	Apple
	Erie	—	2 (24)	—	—
	Franklin	1 (9)	0 (9)	0 (9)	Apple
	Berks (2)	5 (9)	0 (8)	0 (8)	Apple, Pear
	Lycoming	—	0 (27)	—	—
	Allegheny	—	9 (18)	0 (9)	—
	Centre	1 (8)	0 (8)	0 (9)	Apple

## Data Availability

The data presented in this study are available in the Supplementary Material 1: All Count Data.

## Supplementary Materials

The following are available online at https://www.mdpi.com/2075-4450/12/3/258/s1, Supplementary Material 1: All Count Data; Supplementary Material 2: Exemplary Analysis Code.

## References

[1] Hoebeke E.R., Carter M.E. (2003). A polyphagous plant pest from Asia newly detected in North America. Proc. Entomol. Soc. Washingt..

[2] Rice K.B., Bergh C.J., Bergmann E.J., Biddinger D.J., Dieckhoff C., Dively G., Fraser H., Gariepy T., Hamilton G., Haye T. (2014). Biology, ecology, and management of brown marmorated stink bug (Hemiptera: Pentatomidae). J. Integr. Pest Manag..

[3] Lee D., Short B.D., Joseph S.V., Bergh J.C., Leskey T.C. (2013). Review of the biology, ecology, and management of *Halyomorpha halys* (Hemiptera: Pentatomidae) in China, Japan, and the Republic of Korea. Environ. Entomol..

[4] Arakawa R., Namura Y. (2002). Effects of temperature on development of three *Trissolcus* spp. (Hymenoptera: Scelionidae), egg parasitoids of the brown marmorated stink bug, *Halyomorpha halys* (Hemiptera: Pentatomidae). Entomol. Sci..

[5] Talamas E.J., Johnson N.F., Buffington M. (2015). Key to Nearctic species of *Trissolcus* Ashmead (Hymenoptera, Scelionidae), natural enemies of native and invasive stink bugs (Hemiptera, Pentatomidae). J. Hymenopt. Res..

[6] Yang Z.-Q., Yao Y.-X., Qiu L.-F., Li Z.-X. (2009). A new species of *Trissolcus* (Hymenoptera: Scelionidae) parasitizing eggs of *Halyomorpha halys* (Heteroptera: Pentatomidae) in China with comments on its biology. Ann. Entomol. Soc. Am..

[7] Scaccini D., Falagiarda M., Tortorici F., Martinez-Sañudo I., Tirello P., Reyes-Domínguez Y., Gallmetzer A., Tavella L., Zandigiacomo P., Duso C. (2020). An insight into the role of *Trissolcus mitsukurii* as biological control agent of *Halyomorpha halys* in Northeastern Italy. Insects.

[8] Botch P.S., Delfosse E.S. (2018). Host-acceptance behavior of *Trissolcus japonicus* (Hymenoptera: Scelionidae) reared on the invasive *Halyomorpha halys* (Heteroptera: Pentatomidae) and nontarget species. Environ. Entomol..

[9] Milnes J.M., Beers E.H. (2019). *Trissolcus japonicus* (Hymenoptera: Scelionidae) causes low levels of parasitism in three North American pentatomids under field conditions. J. Insect Sci..

[10] Lara J.R., Pickett C.H., Kamiyama M.T., Figueroa S., Romo M., Cabanas C., Bazurto V., Strode V., Briseno K., Lewis M. (2019). Physiological host range of *Trissolcus japonicus* in relation to *Halyomorpha halys* and other pentatomids from California. BioControl.

[11] Abram P.K., Talamas E.J., Acheampong S., Mason P.G., Gariepy T.D. (2019). First detection of the samurai wasp, *Trissolcus japonicus* (Ashmead) (Hymenoptera, Scelionidae), in Canada. J. Hymenopt. Res..

[12] Hedstrom C., Lowenstein D., Andrews H., Bai B., Wiman N. (2017). Pentatomid host suitability and the discovery of introduced populations of *Trissolcus japonicus* in Oregon. J. Pest Sci..

[13] Talamas E., Herlihy M.V., Dieckhoff C., Hoelmer K.A., Buffington M., Bon M.-C., Weber D.C. (2015). *Trissolcus japonicus* (Ashmead) (Hymenoptera, Scelionidae) emerges in North America. J. Hymenopt. Res..

[14] Herlihy M.V., Talamas E.J., Weber D.C. (2016). Attack and success of native and exotic parasitoids on eggs of *Halyomorpha halys* in three maryland habitats. PLoS ONE.

[15] Milnes J.M., Wiman N.G., Talamas E.J., Brunner J.F., Hoelmer K.A., Buffington M.L., Beers E.H. (2016). Discovery of an exotic egg parasitoid of the brown marmorated stink bug, *Halyomorpha halys* (Stål) in the Pacific Northwest. Proc. Entomol. Soc. Washingt..

[16] Stahl J., Tortorici F., Pontini M., Bon M.-C., Hoelmer K., Marazzi C., Tavella L., Haye T. (2019). First discovery of adventive populations of *Trissolcus japonicus* in Europe. J. Pest Sci..

[17] Jarrett B.J.M., Pote J., Talamas E., Gut L., Szucs M. (2019). The Discovery of *Trissolcus japonicus* (Hymenoptera: Scelionidae) in Michigan. Gt. Lakes Entomol..

[18] Kaser J.M., Akotsen-Mensah C., Talamas E.J., Nielsen A.L. (2018). First report of *Trissolcus japonicus* parasitizing *Halyomorpha halys* in North American agriculture. Fla. Entomol..

[19] Quinn N.F., Talamas E.J., Acebes-Doria A.L., Leskey T.C., Bergh J.C. (2019). Vertical sampling in tree canopies for *Halyomorpha halys* (Hemiptera: Pentatomidae) life stages and its egg parasitoid, *Trissolcus japonicus* (Hymenoptera: Scelionidae). Environ. Entomol..

[20] Holthouse M., Schumm Z., Talamas E., Spears L., Alston D. (2020). Surveys in northern Utah for egg parasitoids of *Halyomorpha halys* (Stål) (Hemiptera: Pentatomidae) detect *Trissolcus japonicus* (Ashmead) (Hymenoptera: Scelionidae). Biodivers. Data J..

[21] Sabbatini Peverieri G., Talamas E., Bon M.C., Marianelli L., Bernardinelli I., Malossini G., Benvenuto L., Roversi P.F., Hoelmer K. (2018). Two Asian egg parasitoids of *Halyomorpha halys* (Stål) (Hemiptera, Pentatomidae) emerge in northern Italy: *Trissolcus mitsukurii* (Ashmead) and *Trissolcus japonicus* (Ashmead) (Hymenoptera, Scelionidae). J. Hymenopt. Res..

[22] Quinn N.F., Talamas E.J., Leskey T.C., Bergh J.C. (2019). Sampling methods for adventive *Trissolcus japonicus* (Hymenoptera: Scelionidae) in a wild tree host of *Halyomorpha halys* (Hemiptera: Pentatomidae). J. Econ. Entomol..

[23] Lowenstein D.M., Andrews H., Hilton R.J., Kaiser C., Wiman N.G. (2019). Establishment in an introduced range: Dispersal capacity and winter survival of *Trissolcus japonicus*, an adventive egg parasitoid. Insects.

[24] Tillman G., Toews M., Blaauw B., Sial A., Cottrell T., Talamas E., Buntin D., Joseph S., Balusu R., Fadamiro H. (2020). Parasitism and predation of sentinel eggs of the invasive brown marmorated stink bug, *Halyomorpha halys* (Stål) (Hemiptera: Pentatomidae), in the southeastern US. Biol. Control.

[25] Johnson N.F. (1984). Systematics of Nearctic Telenomus: Classification and Revisions of the Podisi and Phymatae Species Groups (Hymenoptera: Scelionidae).

[26] R. Core Team (2018). R: A Language and Environment for Statistical Computing.

[27] Lenth R. Emmeans: Estimated Marginal Means, Aka Least-Squares Means. https://rdrr.io/cran/emmeans/man/emmeans-package.html.

[28] Todd J.H., Pearce B.M., Barratt B.I.P. (2021). Using qualitative food webs to predict species at risk of indirect effects from a proposed biological control agent. BioControl.

[29] Lowenstein D.M., Andrews H., Mugica A., Wiman N.G., Nielsen A. (2019). sensitivity of the egg parasitoid *Trissolcus japonicus* (Hymenoptera: Scelionidae) to field and laboratory-applied insecticide residue. J. Econ. Entomol..

[30] Haye T., Moraglio S.T., Stahl J., Visentin S., Gregorio T., Tavella L. (2020). Fundamental host range of *Trissolcus japonicus* in Europe. J. Pest Sci..

[31] Zhang J., Zhang F., Gariepy T., Mason P., Gillespie D., Talamas E., Haye T. (2017). Seasonal parasitism and host specificity of *Trissolcus japonicus* in northern China. J. Pest Sci..

