# Supplementary material for: Survey for Adventive Populations of the Samurai Wasp, Trissolcus japonicus (Hymenoptera: Scelionidae) in Pennsylvania at Commercial Fruit Orchards and the Surrounding Forest
Source: Insects. 2021 Mar 19;12(3):258. doi: 10.3390/insects12030258 (PMC8003256; doi:10.3390/insects12030258)
Supplement: Supplementary file 1 [file insects-12-00258-s001.zip › insects-1101335-supplementary/insects-1101335-conversion/Supplementary Material 2/Peterson Exemplary R Code .rtf]

#Survey for adventive populations of the samurai wasp,# Trissolcus japonicus (Hymenoptera: Scelionidae) in Pennsylvania at commercial fruit orchards# and the surrounding forest.#Exemplary Code for All Analyses Conducted#All analyses  in the study were conducted using the following methods#Created by Hillary M. Peterson, February 9, 2021rm(list = ls())YSC <- read.csv("2018 All Wasps YSC Comparisons TJ Survey.csv")library(multcomp)library(lme4)library(lmerTest)library(emmeans)library(ggplot2)library(car)library(tidyverse)library(optimx)library(parallel)library(minqa)county <- YSC$County..Location.season <- YSC$Seasonalityhabitat <- YSC$Habitatdays <- YSC$Days.Exposedeuschisti <- YSC$Trissolcus.euschistibrochymenae <- YSC$Trissolcus.brochymenaejaponicus <- YSC$Trissolcus.japonicuspodisi <- YSC$Telenomus.podisiwasps <- YSC$All.Wasps#Begin with a simple plot to ensure that data loaded properlyplot=ggplot(data=YSC,aes(x=habitat, y=wasps)) #plotting the summary data, colored with species identity plot=plot+geom_bar(stat='identity') # making a bar chart, not summarizing here (identity)plot#Comparison of ALL wasps:wasps.model.1 <- glmer.nb(wasps ~ season*habitat + (1|county) + offset(log(days)), data = YSC)Anova(wasps.model.1)#Significant differences between the season and habitat with no interaction.#The Tukey’s method for comparing estimated marginal means is used to compare values# with significant differences across the treatments.emmeans(wasps.model.1,specs=pairwise~habitat,type="response")emmeans(wasps.model.1,specs=pairwise~season,type="response")#The models below demonstrate that there is not enough power in the analysis to compare# Trissolcus japonicus across the season or habitat, as these data do not converge.#Comparison of Trissolcus japonicus across the seasonalities and habitats.japonicus.model.1 <- glmer.nb(japonicus ~ season*habitat + (1|county) + offset(log(days)), data = YSC)#An important note:# In the 2019 analysis, in a few cases different optimizers were needed in order to# have the analyses converge. The function below was used for the trials of differenct# optimizers. This code was written by Joshua Nugent. For more information on convergence:?allFit?lme4::convergence# https://joshua-nugent.github.io/allFit/################## ALL FIT FUNCTION ##############################model name in this example is example.modeldiff_optims <- allFit(example.model, maxfun = 1e5, parallel = 'multicore', ncpus = detectCores())is.OK <- sapply(diff_optims, is, "merMod")diff_optims.OK <- diff_optims[is.OK]lapply(diff_optims.OK,function(x) x@optinfo$conv$lme4$messages)convergence_results <- lapply(diff_optims.OK,function(x) x@optinfo$conv$lme4$messages)working_indices <- sapply(convergence_results, is.null)if(sum(working_indices)==0){  print("No algorithms from allFit converged.")  print("You may still be able to use the results, but proceed with extreme caution.")  first_fit <- NULL} else {  first_fit <- diff_optims[working_indices][[1]]}first_fitAnova(first_fit)
